# Integrative Analysis of Whole Genome Sequencing and Phenotypic Resistance Toward Prediction of Trimethoprim-Sulfamethoxazole Resistance in *Staphylococcus aureus*

**DOI:** 10.3389/fmicb.2020.607842

**Published:** 2021-01-14

**Authors:** Dennis Nurjadi, Elfi Zizmann, Quan Chanthalangsy, Klaus Heeg, Sébastien Boutin

**Affiliations:** Department of Infectious Diseases, Medical Microbiology and Hygiene, Heidelberg University Hospital, Heidelberg, Germany

**Keywords:** AMR prediction, *Staphylococcus aureus*, co-trimoxazole, trimethoprim resistance, sulfamethoxazole resistance, WGS, antifolate antibiotics

## Abstract

As whole genome sequencing is becoming more accessible and affordable for clinical microbiological diagnostics, the reliability of genotypic antimicrobial resistance (AMR) prediction from sequencing data is an important issue to address. Computational AMR prediction can be performed at multiple levels. The first-level approach, such as simple AMR search relies heavily on the quality of the information fed into the database. However, AMR due to mutations are often undetected, since this is not included in the database or poorly documented. Using co-trimoxazole (trimethoprim-sulfamethoxazole) resistance in *Staphylococcus aureus*, we compared single-level and multi-level analysis to investigate the strengths and weaknesses of both approaches. The results revealed that a single mutation in the AMR gene on the nucleotide level may produce false positive results, which could have been detected if protein sequence analysis would have been performed. For AMR predictions based on chromosomal mutations, such as the *folP* gene of *S. aureus*, natural genetic variations should be taken into account to differentiate between variants linked to genetic lineage (MLST) and not over-estimate the potential resistant variants. Our study showed that careful analysis of the whole genome data and additional criterion such as lineage-independent mutations may be useful for identification of mutations leading to phenotypic resistance. Furthermore, the creation of reliable database for point mutations is needed to fully automatized AMR prediction.

## Introduction

The reliable detection and prediction of antimicrobial resistance is an on-going issue in the era of antimicrobial resistance (AMR) with significant clinical implications. Infections with multidrug-resistant organisms (MDRO) are difficult to treat and often results in increased morbidity, mortality and prolonged length of hospital stay (Cassini et al., 2019). Phenotypic detection of AMR, i.e., culture-based, is still the current gold standard in microbiological diagnostics. Although significant advances in whole genome sequencing have brought major breakthroughs for genome-based AMR prediction, this method is not yet completely accurate (Ellington et al., 2017; Hendriksen et al., 2019).

Genotypic AMR prediction relies heavily on the quality of the available database (Hendriksen et al., 2019). On top, data analysis can be performed on multiple levels, such as nucleotide alignment, protein sequence alignment and with multiple levels of resolution; read or assembly based. Whereas the identification of AMR genes is quite simple, the identification of resistance determinants due to genetic variants is more challenging (Doyle et al., 2020). Instead of identifying the presence or absence of a particular gene, the analysis would have to differentiate the non-synonymous polymorphism to extrapolate the protein sequences and discriminate between susceptible and resistant isolates. Misclassification of resistant isolates as susceptible isolates, i.e., false negative, would have profound clinical implications and therefore should be avoided.

In order to identify the strengths, weaknesses and limitations of computational analysis in detecting AMR, we performed a comparative analysis of resistance determinants for trimethoprim-sulfamethoxazole (TMP-SMZ or SXT) in the clinically relevant pathogen, *Staphylococcus aureus*. SXT belongs to the antifolate antibiotic. Its antimicrobial activity is based on the competitive inhibition of two successive essential enzymes in the bacterial folic acid pathway, which inhibits thymidine synthesis and subsequently, DNA synthesis (Bushby and Hitchings, 1968). TMP-SMZ resistance in *S. aureus* is particularly interesting for AMR prediction due to the different resistance mechanisms (Huovinen, 2001). TMP resistance in *S. aureus* is predominantly mediated by the acquisition of an extra-chromosomal dihydrofolate reductase (DHFR) encoding genes [*dfrA (synonym dfrS1)*, *dfrG*, *dfrK*] (Nurjadi et al., 2014, 2015), whereas the underlying mechanisms for SMZ resistance are mutations on the chromosomal dihydropteroate synthase (DHPS) encoding gene (*folP*) (Griffith et al., 2018). In this study, we compared the phenotypic-genotypic resistance concordance of sequenced clinical *S. aureus* isolates from our hospital to investigate the limitations of single-level analysis of the genome data and the advantages of multi-level analysis for AMR prediction.

## Materials and Methods

### Study Samples

Sequenced clinical *S. aureus* isolates (*n* = 242) from the routine microbiological diagnostics of the Heidelberg University Hospital between 2018 and 2020 are included in this study. To ensure uniformity in testing procedure, the phenotypic AST was repeated using the same method for this study.

### Phenotypic Antimicrobial Susceptibility Testing (AST)

Phenotypic AST was performed by the Kirby-Bauer disk diffusion method on Mueller-Hinton Agar (bioMérieux GmbH, Germany) according to the EUCAST recommendations. Three antimicrobial agents were tested, trimethoprim (5 μg, BD Diagnostics, Germany), sulfonamide (300 μg, Oxoid, Germany), and trimethoprim-sulfamethoxazole (BD Diagnostics, Germany). The agar plates were read after 18–20 h incubation at 37°C without CO_2_. Zone of inhibition (in mm) for TMP (<14 mm = *R*) and SXT (<14 mm = *R*; 14–16 mm = *I*; ≥17 mm = *S*) were interpreted according to the EUCAST clinical breakpoints (v10.0) and for SMZ (≤12 mm = *R*; 13–16 = *I* and ≥17 mm = *S*) according to the CLSI cut-off.

### Whole Genome Sequencing and Data Analysis

Genomic DNA was extracted from overnight bacterial culture using the DNeasy Blood and Tissue Mini kit (QIAGEN GmbH, Germany). Standard genomic library was prepared from the bacterial DNA and sequenced with the Illumina MiSeq platform (2 × 300 bp paired end), as described elsewhere (Klein et al., 2020). For quality control, raw sequences were trimmed using Sickle 1.33 (parameters, *q* > 30; 1 > 45). Obtained contigs were curated for length (>1000 bp) and coverage (>10×). Sequences are available under the BioProject-Numbers PRJNA561696 and PRJNA637212. Sequences were annotated using Prokka 1.14.1 (Seemann, 2014) (based on Genetic Code Table 11). Resistance genes were found using Abricate 0.8.13 with the databases from ResFinder, NCBI, CARD, ARG-ANNOT (Zankari et al., 2012; Gupta et al., 2014; Jia et al., 2017; Feldgarden et al., 2019), to identify potential variants in the DHFR genes. For the DHPS gene, the region of interest was extracted from the assembly with Samtools (Etherington et al., 2015) and aligned with MAFFT (Nakamura et al., 2018). Unique representative sequences were obtained with CD-Hit (Li and Godzik, 2006) (100% identity) and SNPs were then called with snp-sites (Page et al., 2016).

## Results

Altogether 242 *S. aureus* isolates were analyzed in this study. The clinical isolates were collected through the routine microbiological diagnostic. For comparability and uniformity in phenotypic AST, all isolates were re-tested for this study using the agar diffusion method. 38.4% (93/242) were phenotypically resistant to TMP, 2% (5/242) exhibited reduced susceptibility to SMZ (two were resistant and three were intermediate), and 3.3% (8/242) exhibited reduced susceptibility to SXT (two resistant and six intermediate).

### Trimethoprim Resistance

**FIGURE 1:**
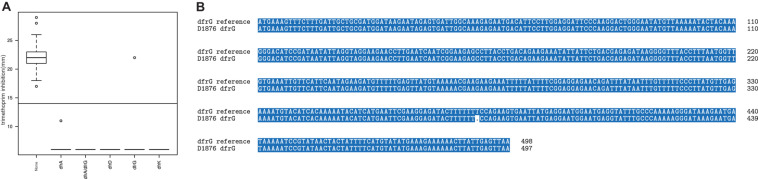
Extra chromosomal DHFR predicted trimethoprim resistance. **(A)** zone of inhibition of trimethoprim (in mm) grouped by the DHFR variants. One isolate (D1876) carrying *dfrG* exhibited a susceptible phenotype. **(B)** Nucleotide sequence of the *dfrG* in D1876 showed a thymine deletion at position 381 compared to the reference *dfrG* sequence.

Out of the 93 phenotypically TMP-resistant isolates, all harbored known AMR genes (*dfrA*, *dfrG*, *dfrD*, *dfrK*). The majority (61/93; 65.6%) harbored *dfrG*, 27/93 (29%) harbored *dfrA*, 1/93 (1%) harbored *dfrD*, 1/93 (1%) had *dfrK* and 3/93 (3.2%) isolates had more than one *dfr* genes (*dfrA* and *dfrG*). Screening for known TMP AMR genes from the draft genome resulted in 62 *dfrG* genes, but only 61 correlated with the phenotypical TMP resistance (Figure 1A). We observed two outliers in the comparison between genotypic and phenotypic resistance. The first outlier harbors the *dfrG* AMR gene but was phenotypically susceptible to TMP. Alignment of the of the DHFR protein sequence of the *dfrG*-positive (DHFR*_*dfrG*_*) and TMP susceptible isolate with the functional DHFR*_*dfrG*_* revealed a frameshift due to a deletion of thymine at position 381 in the DHFR*_*dfrG*_* sequence (Figure 1B), which resulted in a truncation of the DHFR*_*dfrG*_* and possibly a non-functional DHFR*_*dfrG*_* (Supplementary Figure 1). The second outlier, an isolate with *dfrA* exhibited a reduced zone of inhibition (11 mm). The acquisition of extra-chromosomal DHFR encoding gene usually results in high-level resistance to TMP (Dale et al., 1995) and hence no zone of inhibition is expected. According to the EUCAST clinical breakpoint this is still considered resistant, so that this isolate would not be falsely classified by genotypic AMR prediction. We did not find any putative underlying mutation in the gene or promoter region, which could explain this deviation.

### Sulfamethoxazole Resistance

**FIGURE 2:**
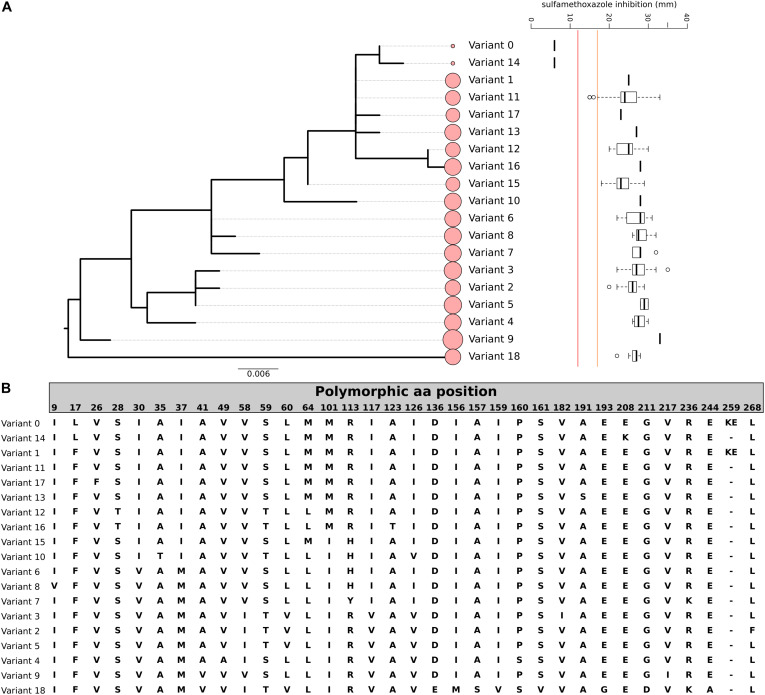
Genomic variation of DHPS among our study cohort. **(A)** Phylogenetic relationship of the 19 variants of DHPS (var. 0–18) and the corresponding SMZ resistance (red line indicates the CLSI cut-off for SMZ resistance, orange line indicates the cut-off for SMZ susceptibility, intermediate range between red and orange line, the size of the circles indicate the number of isolates in the variant cluster). The tree is based on the nucleotide sequence and rooted at midpoint. **(B)** Amino acid sequence of the 19 DHPS variants detected. Only polymorphic sites are displayed. The order corresponds to the phylogenetic tree order.

Sulfamethoxazole resistance in our study isolates were relatively rare; only two isolates exhibited a high-level resistant phenotype (no zone of inhibition). Since SMZ mutation in *S. aureus* is not mediated by the acquisition of AMR genes, rather mutations in the chromosomal DHPS, we performed a protein sequence alignment of the DHPS sequence to identify variations and mutations. We found 19 DHPS variants; a phylogeny based on the amino acid profile of the DHPS is displayed in Figure 2. Only isolates belonging to DHPS_*var0*_ and DHPS_*var14*_ were phenotypically resistant to SMZ, with known functional primary DHPS mutation at the position F17L. DHPS_*var14*_ had an additional duplication of at position 257 (KE257_Dup), which has been described as a secondary mutation conferring SMZ resistance in *S. aureus* (Griffith et al., 2018). However, this duplication alone may not suffice to encode high-level resistance as another variant, DHPS_*var1*_; exhibiting the same duplication was not phenotypically resistant to SMZ. The three isolates with an intermediate resistance phenotype had the same DHPS variant (DHPS_*var11*_). Since the majority of the isolates belonging to this cluster were not phenotypically resistant to SMZ, it is unlikely that DHPS_*var11*_ is responsible for reduced susceptibility toward SMZ in this cluster. Our analysis clearly highlights the limitation of this method that genotypic AMR prediction of mutation-mediated resistance is restricted to known resistance mechanisms.

### SXT (Combined TMP-SMZ) Resistance

**FIGURE 3:**
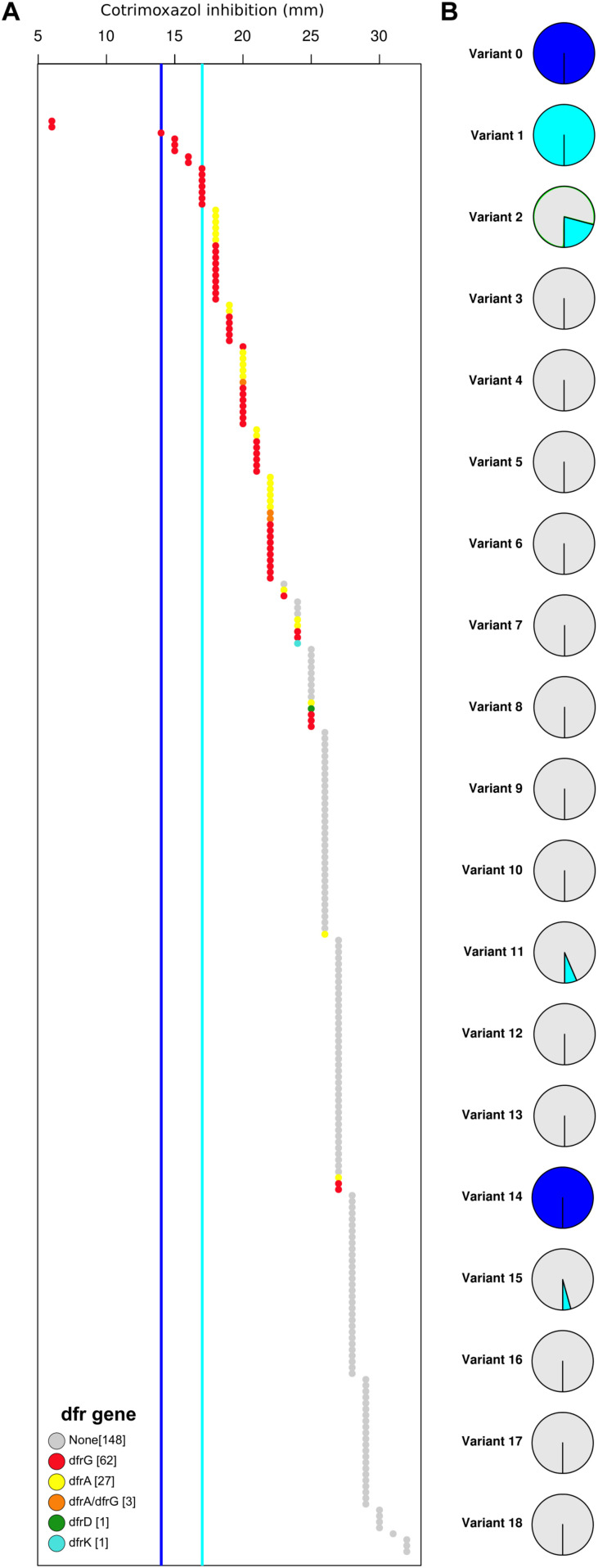
High-level co-trimoxazole (SXT) resistance is the result of a DHPS mutation (F17L) and the acquisition of an extra-chromosomal *dfr* gene. **(A)** All strains are represented in an increasing order based on the zone of inhibition of co-trimoxazole (in mm). Color represent the extra-chromosomal DHFR variant (*dfr* gene). Dark blue line indicates cut-off for SXT resistance, Light blue line indicates cut-off for SXT susceptibility. **(B)** The percentage of resistant (dark blue), intermediate (light blue), and sensitive (gray) strains are displayed as a pie-chart for each DHPS variant.

Overall, only two isolates showed a high-level SXT resistant phenotype (no inhibition zone), and both isolates harbor a *dfr* gene and the F17L mutation in the DHPS protein sequence. Other isolates with phenotypically reduced susceptibility to SXT harbor extra-chromosomal *dfr* genes. There was no clear correlation between DHPS variants and reduced susceptibility to the drug combination. Although the presence of extrachromosomal DHFR encoding *dfr* genes did not result in high-level resistance to the combined drug, we observed a clear correlation between the presence of *dfr* genes and the reduction in the diameter of the zone of inhibition for SXT (Figure 3). Our analysis strongly indicated that resistances to both components were necessary for high-level resistance to the combined drug. Moreover, TMP resistance determinants may have a more significant influence on the SXT resistance phenotype than variations in the DHPS protein sequence.

### Genetic Background and Resistance Determinants

Our study isolates were clonally diverse as displayed by the minimum spanning tree based on the core genome (Supplementary Figure 2A). Most of the DHPS variants correlate well with the MLST (Supplementary Figures 2A,B), which suggest that the major clusters of DHPS variants might be a clonal characteristic. Furthermore, the two SMZ-resistant DHPS variants belong to different MLSTs (ST8 and ST241), which indicate that functional mutations leading to phenotypic resistance occur independent of the genetic lineage and might be a useful criterion for computational analysis of mutation-based resistance determinants (Supplementary Figure 2B). The presence of *dfr* genes is dispersed among the genetic lineages (Supplementary Figure 2C).

## Discussion

*In silico* AMR detection and prediction is theoretically a powerful and easy to implement tool for molecular microbiology diagnostic with several limitations. On a positive note, our data suggest that current AMR database is reliable enough to correctly predict all TMP and SMZ resistance phenotype in 242 study isolates. Only two isolates were falsely classified as TMP resistant by genotypic AMR prediction. Indeed, false positivity would only be considered a minor error as this would not lead to therapy failure due to non-susceptibility, which would be the case for false susceptible prediction. Nevertheless, our study demonstrated that single-level analysis may not be sufficient and that a multi-level analysis should be performed to improve accuracy of molecular AMR prediction.

The simplest tool for AMR prediction is the detection of AMR gene presence or absence, which was the case for TMP resistance in our study. Consistent with the literature, most of the strains carrying an extra *dfr* gene (*dfrA*, *dfrD*, *dfrG*, *dfrK*) displayed a high-level resistant phenotype (Dale et al., 1995; Nurjadi et al., 2014, 2015). However, two isolates showed only reduced susceptibility (low-level resistance) or even a sensitive phenotype. The low-level resistant strain carried a *dfrA* gene without any mutations in the coding or the promoter region explaining the phenotype, which was unexpected since the presence of *dfrA* (DHFR S1) is linked with high-level TMP resistance (Dale et al., 1995). We hypothesize that this decrease in resistance may be a result of lower expression of the *dfrA* gene, which could only be validated by transcriptional analysis. The second strain with a TMP susceptible phenotype carried a *dfrG* gene, which generally also confers high-level resistance (Sekiguchi et al., 2005). However, the analysis at the nucleotide level showed a frameshift mutation at position 381, leading to a truncated protein. Interestingly, by interrogating the different AMR database, the identity score of this variant to the wild type is 99,81% which in most cases would be considered as the presence of a functional variant. Furthermore, the deletion of one specific thymidine in a homopolymer region containing seven consecutive thymidine can easily be interpreted as a sequencing or assembly errors because those regions are known to be prone to errors (Ross et al., 2013; Heydari et al., 2019). In our case, by only considering AMR gene presence and absence, this mutation, being the only mutation present in the gene and promoter region, may have been overlooked and the isolate would have been falsely classified as resistant. This is a good example, how a single-level computational AMR prediction based on the nucleotides and gene presence only may lead to false results and that additional protein analysis may provide additional useful information.

Besides gene acquisition, chromosomal gene mutations can often mediate resistance to antibiotics. In *S. aureus*, SMZ resistance is mediated by mutations in the DHPS-encoding gene, *folP*. F17L, S18L, and T51M, are considered as primary DHPS mutations conferring SMZ resistance, whereas E208K and KE257_Dup are considered as secondary mutations (Griffith et al., 2018). Non-synonymous point mutations are often associated in the literature with resistance potential, but our phenotypical data showed that only one mutation (F17L) is clearly associated with the resistant phenotype, which is consistent with published data. The sole presence of the secondary mutation KE257_Dup did not lead to phenotypic resistance to SMZ By characterizing and alignment of the DHPS protein sequences, we could identify 19 allelic variations in our study isolates. The DHPS variations observed in our study relates mostly to the diversity of strains (i.e., natural polymorphisms), and we observed a good concordance between the DHPS variants and the genetic lineage on MLST level. Our data indicate that functional mutations occur independently of clonal lineage and that this characteristic may be useful to increase the accuracy of computational prediction of mutation-based AMR and should be included in the database. In addition, phenotypic AMR is integral and should be performed to validate mutation-mediated resistance.

Antimicrobial resistance prediction is entirely dependent on the quality of gene databases. Most of the standard software or pipelines are currently using the same databases (CARD, NCBI, ResFinder, ARG-ANNOT, etc.) either independently or combined. While the teams working on updating those databases do remarkable work, it is not easy to update the database in real-time. In our case, the database was up to date, and the *dfr* genes were present in the database. However, the identity threshold needs to be set-up really high (100%) to make sure that no false resistant will be predicted. At the same time, we need to be cautious of false negative reports, which may result from overseeing functional mutants. Similar to the variants of the DHPS-encoding genes, not every DHFR variant is functional. Therefore, for NGS based clinical diagnostic, we will need an extensive database at the nucleotide and protein levels of the genes of interest to ensure a correct and reliable prediction. Until then, phenotypic AST will remain as a reference method. The advances in WGS associated with the classical phenotypic AST will help build an accurate database by feeding both the draft genomes and the resistance phenotype in a machine-learning algorithm to highlight the genes variants and hot-spot genomic region associated with the AMR (Macesic et al., 2017; Aytan-Aktug et al., 2020; Kim et al., 2020). Of course, the harmonization of phenotypic AST is essential to minimize interpretation or technical errors and deviations, which may be associated with specific testing methods.

One major issue for genotypic AMR prediction is the detection and interpretation of “intermediate” resistance. Our data demonstrated that the presence of DHFR genes (*dfrA* and *dfrG*) may had some effect on the SXT susceptibility (Figure 3). Isolates, which harbor extra-chromosomal DHFR genes exhibited smaller inhibition zones than those without. From the clinical point of view, the antibiotic substance can be used to treat infections with bacteria with an intermediate resistance phenotype if the *in vivo* concentration at the infection localization can be reached by standard or high-dose therapy regimen (Kahlmeter and Committee, 2017; Wantia et al., 2020). The intermediate resistance phenotype is an expression of reduced susceptibility to an antibiotic substance. However, in most cases the underlying mechanism for intermediate resistance is often unclear and cannot be explained by acquisition of AMR gene or chromosomal mutations. This aspect should be explored and clarified prior to introduction of genotypic AMR prediction in the clinical diagnostic setting.

Our studies have limitations. Although, we could demonstrate that *folP* variants go hand in hand with the genetic lineage, we still cannot be sure whether our analysis represents the whole spectrum of DHPS variants in the general *S. aureus* population. Several of the strains exhibit intermediate resistance phenotype, i.e., reduced susceptibility, but we did not find any strong indication if this phenotype is associated with a particular DHPS variant. Further studies are needed to investigate the underlying mechanism of intermediate resistance. The *in silico* AMR prediction to the combined drug (SXT) in *S. aureus* remains challenging. Only isolates with an extra-chromosomal DHFR (*dfr* genes) and the primary mutation F17L, exhibited high-level resistance to SXT. The presence of *dfrA* or *dfrG* alone does not confer high-level resistance to SXT. Nevertheless, we could demonstrate a clear correlation between the presence of these genes and reduced susceptibility to SXT.

## Conclusion

Taken together, using SXT resistance in *S. aureus*, we demonstrated that genotypic AMR prediction using the current tools and database is reliable. However, a multi-level analysis approach by incorporating nucleotide and allelic variance, and protein sequence analysis may be useful to increase the accuracy of genotypic AMR prediction and concordance between genotypic and phenotypic resistance. On top, harmonized phenotypic resistance data should be incorporated into AMR databases to increase accuracy of computational AMR prediction. For the time being, *in silico* AMR prediction is not yet perfect and phenotypic resistance testing remains indispensable.

## Data Availability Statement

The datasets presented in this study can be found in online repositories. The names of the repository/repositories and accession number(s) can be found below: https://www.ncbi.nlm.nih.gov/ (PRJNA561696 and PRJNA637212).

## Author Contributions

DN, KH, and SB designed the study. DN and SB drafted the manuscript and all authors finalized the manuscript. EZ and QC performed the phenotypical testing for TMP, SMZ, and SXT. SB performed the WGS analysis. DN, EZ, and QC interpreted and analyzed the phenotypical resistance data. All authors contributed to the article and approved the submitted version.

## Conflict of Interest

The authors declare that the research was conducted in the absence of any commercial or financial relationships that could be construed as a potential conflict of interest.

## Supplementary Material

Supplementary Figure 1 Amino acid sequence of a truncated DHFR*_*dfrG*_* (D1876) due to a nucleotide deletion at position 381 of the *dfrG* resulting in a trimethoprim susceptible phenotype.

Supplementary Figure 2 Minimum spanning tree (MST) of our study cohort. The MST is calculated using all 67571 polymorphic sites across the core-genome and with the MSTV algorithm from the software GrapeTree Strains are color-coded based either on **(A)** their MLSTs, **(B)** their DHPS variants, **(C)** their *dfr* genes. The arrows in **(B)** indicate the two high-level SMZ resistant *S. aureus* isolates harboring the F17L mutation of the chromosomal DHPS.
