# Supplementary material for: Integrative Analysis of Whole Genome Sequencing and Phenotypic Resistance Toward Prediction of Trimethoprim-Sulfamethoxazole Resistance in Staphylococcus aureus
Source: Front Microbiol. 2021 Jan 14;11:607842. doi: 10.3389/fmicb.2020.607842 (PMC7840696; doi:10.3389/fmicb.2020.607842)
Supplement: Supplementary Figure 1 — Amino acid sequence of a truncated DHFRdfrG (D1876) due to a nucleotide deletion at position 381 of the dfrG resulting in a trimethoprim susceptible phenotype. [file Data_Sheet_1.pdf]

```
dfrG reference   MKVSLIAAMDKNRVIGKENDIPWRIPKDWEYVKNTTKGHPIILGRKNLESIGRALPDRRNIILTRDKGFTFNGCEIVHSIEDVFELCKNEEEIFIFGGEQIYNLFFPYVE   110
D1876 dfrG       MKVSLIAAMDKNRVIGKENDIPWRIPKDWEYVKNTTKGHPIILGRKNLESIGRALPDRRNIILTRDKGFTFNGCEIVHSIEDVFELCKNEEEIFIFGGEQIYNLFFPYVE   110

dfrG reference   KMYITKIHHEFEGDTFFPEVNYEEWNEVFAQKGIKNDKNPYNYYFHVYERKNLLSX   166
D1876 dfrG       KMYITKIHHEFEGDTFFQKX....................................   130
```

Supplementary figure 1. amino acid sequence of a truncated $DHFR_{dfrG}$ (D1876) due to a nucleotide deletion at position 381 of the *dfrG* resulting in a trimethoprim susceptible phenotype.

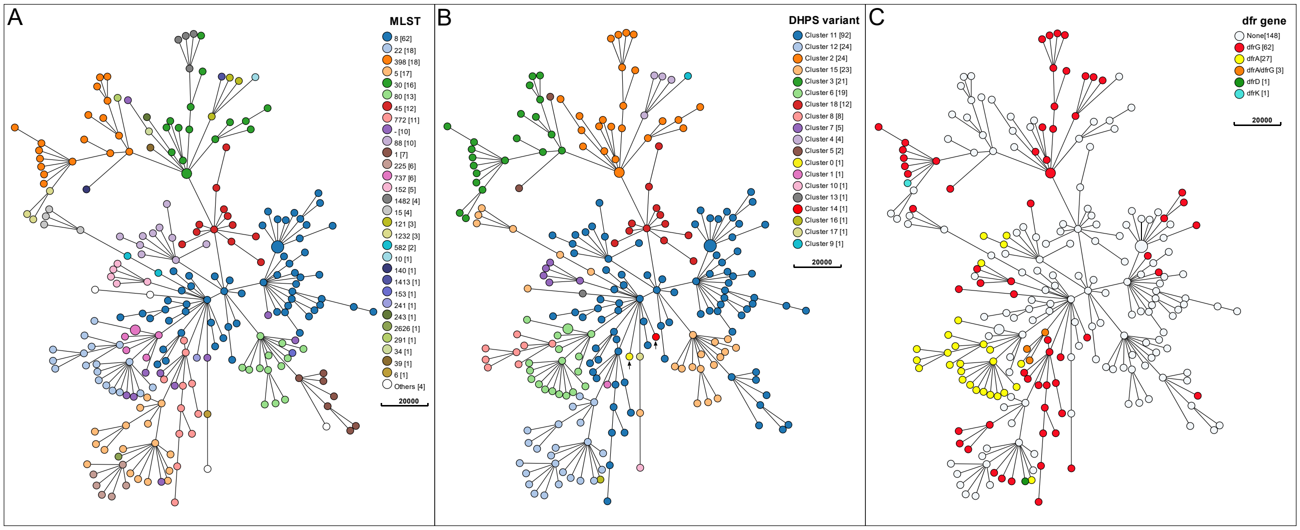

Supplementary figure 2. Minimum spanning tree (MST) of our study cohort. The MST is calculated using all 67571 polymorphic sites across the core-genome and with the MSTV" algorithm from the software grapetree Strains are color-coded based either on A) their MLSTs, B) their DHPS variants, C) their *dfr* genes. The arrows in panel B indicate the two high-level SMZ resistant *S. aureus* isolates harboring the F17L mutation of the chromosomal DHPS.
